# Chaihu Shugan prevents cholesterol gallstone formation by ameliorating the microbiota dysbiosis and metabolic disturbance in mice

**DOI:** 10.3389/fphar.2023.1291236

**Published:** 2024-01-31

**Authors:** Wei Wang, Kun Zhang, Bin Liu, Tong Zhou, Yu Tang, Yuliang Li

**Affiliations:** ^1^ Department of Intervention, The Second Hospital of Shandong University, Jinan, China; ^2^ Shanghai Biotree Biotech Co., Ltd, Shanghai, China; ^3^ Department of Geriatrics, National Clinical Research Center for Geriatric Disorders, Xiangya Hospital, Central South University, Changsha, China

**Keywords:** cholesterol gallstone, Chaihu Shugan, obacunone, gut microbiota, gut metabolism

## Abstract

**Introduction:** Cholesterol gallstone (CGS) is a biliary tract disorder requiring treatment in approximately 20% of patients. The efficacy of Chaihu Shugan in preventing CGS recurrence after successful treatment remains uncertain.

**Methods:** We examined the *in vivo* preventive efficacy of Chaihu Shugan using a CGS mouse model and used multi-omics to study the interplay between gut microbiota, metabolism, and gene expression.

**Results:** The intestinal microbiota was severely dysregulated during the formation of CGS, showing a marked decrease in the abundance of beneficial microbiota, especially *Lactobacillus* and *Akkermansia*. Chaihu Shugan prevented CGS formation by restoring the composition of the gut microbiota and reversing the metabolic disturbances caused by dysbiosis. This preventive effect of Chaihu Shugan was paralleled by changes in the expression of metabolism-related genes in the liver. A network pharmacology analysis of Chaihu Shugan revealed that obacunone may be the key active metabolite in regulating bile acid metabolism. Multi-omics and correlation analyses elucidated the interplay between gut microbiota, metabolism, and gene alterations in the dose-dependent effect of Chaihu Shugan.

**Conclusion:** Our data show that Chaihu Shugan can prevent CGS and indicate its mechanisms of action.

## 1 Introduction

Cholesterol gallstone disease (CGS) is a common biliary disorder with a prevalence of up to 20%, of which more than 20% of patients will progress to an asymptomatic or complicated stage (Lammert et al., 2016). CGS risk factors include being female, advanced age, pregnancy, sedentary lifestyle, obesity, hypercholesterolemia, diabetes mellitus, bariatric surgery, and high-fat dietary patterns (Lammert et al., 2016; Chang et al., 2019; Di Ciaula et al., 2019; Haal et al., 2021; Kubica and Balbus, 2021). Many of these factors are associated with metabolic disturbances. The detrimental gut microbiota has also been implicated in promoting CGS by influencing bile acid composition and cholesterol secretion into the biliary system (Hu et al., 2022). It has been observed that modulation of FXR signaling-mediated bile acid metabolism by beneficial gut microbiota such as *Lactobacillus* alleviates CGS (Ye et al., 2022). In our unpublished previous cross-sectional analysis, significant dysbiosis of gut microbiota and metabolism was observed in CGS patients with mutations in gallbladder and liver genes. This interplay between the gut, metabolism, and genes might play an important role in CGS formation.

The use of Chaihu Shugan to prevent post-treatment CGS recurrence was examined in a randomized controlled trial (RCT) conducted by our clinical medicine department. Chaihu Shugan significantly reduced the rate of CGS recurrence compared with the placebo, with minimal adverse effects. Furthermore, Chaihu Shugan has been used to treat a variety of metabolic gastroenterological and hepatobiliary conditions, including chronic gastritis (Qin et al., 2013), irritable bowel syndrome (Hang et al., 2022), hepatic injury (Jia et al., 2017), liver inflammation and hepatic steatosis (Lei et al., 2022), and non-alcoholic fatty liver disease (NAFLD) (Wang et al., 2022a). Scientists have hypothesized that the therapeutic effects of Chaihu Shugan are attributed to its ability to modulate gut microbiota dysbiosis and improve metabolic disorders (Liang et al., 2018; Yang et al., 2022). Based on this premise, we hypothesized that Chaihu Shugan could influence gut microbiota composition, regulate metabolic processes, and prevent CGS formation. We hypothesized that Chaihu Shugan may prevent CGS formation by ameliorating the microbiota dysbiosis and metabolic disturbance. This study aimed to test the above hypothesis. Additionally, we investigated the potential association between gut microbiota, metabolism, gene expression, and CGS formation.

## 2 Materials and methods

### 2.1 Chaihu Shugan preparation

The Chaihu Shugan is a combination of several botanical drugs: 10 g Bupleurum chinense DC, 10 g Citrus × aurantium f. deliciosa (Ten.) M. Hiroe, 10 g Conioselinum anthriscoides ‘Chuanxiong’, 10 g Aconitum carmichaelii Debeaux, 10 g Citrus x aurantiifolia (Christm.) Swingle, 10 g Paeonia lactiflora Pall, 6 g Glycyrrhiza glabra L, 15 g Lysimachia congestiflora Hemsl, 10 g Curcuma longa L and 10 g Lygodium japonicum (Thunb.), which were optimized by a group of Chinese medicine experts. The different botanical drugs that constitute Chaihu Shugan were purchased from Jiangyin Tianjiang Pharmaceutical Co., Ltd. (Jiangyin, China) and authenticated by two experienced pharmacists.

The extraction procedure abide by the type A extract of the ConPhyMP-Guidelines (Heinrich et al., 2022). The botanical drug was boiled with water, filtered, and concentrated into clear paste. Appropriate amount of auxiliary materials was added, dried, crushed and mixed well. Take the above extracts as prescribed and mix them evenly, and 12–40 mesh granules were prepared by the dry method. Check the solubility, moisture and microbial limits according to the inspection routine items. Pack the pellets into small foil bags into 5 g/package, and stored at room temperature. The botanical drugs were dissolved in normal saline before oral gavage.

### 2.2 Cholesterol gallstone mouse model

**FIGURE 1:**
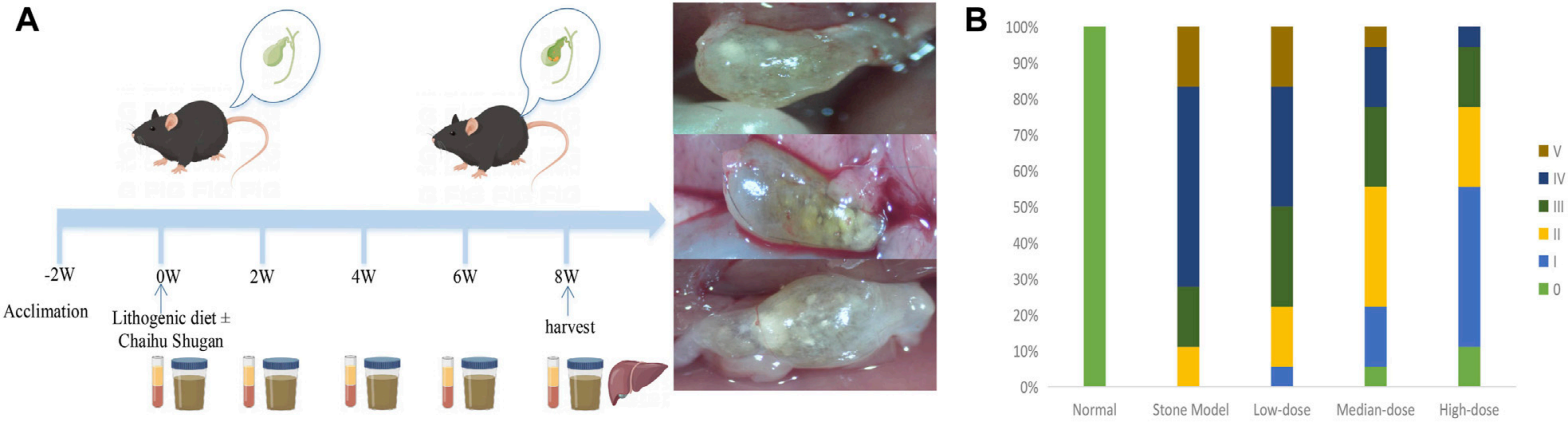
CGS model and preventive efficacy of different Chaihu Shugan doses. **(A)** The CGS model. **(B)** CGS grading of different treatment groups in the eighth week of treatment.

The CGS model was established using 6-week-old C57BL/6 mice. The mice were divided into the CGS model (S), treatment (low-dose, L; medium-dose, M; high-dose, H), and control (C) groups (n = 10 mice per group). The mice were housed in a temperature-controlled room set at 22°C ± 2°C, with a 12-h light/dark cycle (6 a.m.–6 p.m.). A minimum acclimation period of 2 weeks was allowed before commencing the study. Throughout the experimental period, the mice had unrestricted access to water and were fed either normal mouse chow or a lithogenic diet. Specifically, the CGS group received a diet consisting of high levels of cholesterol and fat (10% lipid, 1% cholesterol, and 0.5% cholate; TP 06116F1, Trophic Animal Feed High-tech Co. Ltd., Nantong, China), and the control group was fed a standard diet (LAD0011). The treatment groups were administered a high-cholesterol and high-fat diet and varying doses of Chaihu Shugan (low, medium, and high) via oral gavage. The equivalent dose was calculated based on the body surface area. The mice (20 g) equivalent dose was 0.00162 × human (60 kg) equivalent dose, equal to 0.81 g/kg (Nair et al., 2018). The three-dose gradients were 0.1 ×, 0.5 ×, and 1 × mice equivalent dose (low, medium, and high doses, respectively). Serum and fecal samples were collected at regular intervals every 2 weeks until the eighth week (Figure 1A), when the mice were euthanized, and their livers were collected for RNA-sequencing (RNA-seq). The CGS model grade was assessed using phase contrast and polarized light microscopy images of cholesterol crystallization in the gallbladder bile. The CGS grade was also determined based on staging criteria used by Akiyoshi et al. (Akiyoshi et al., 1986; Zhuang et al., 2021), categorizing CGS from Grades 0 (no gallstones) to V.

### 2.3 Total DNA extraction and 16s rRNA sequencing

TIANamp Stool DNA Kit (Cat#, DP328, Tiangen Biotech Co. Ltd., Beijing, China) was used to extract total DNA from the fecal flora in the collected stool samples according to the manufacturer’s instructions. The quantity and quality of extracted DNA were measured using a NanoDrop spectrophotometer (Thermo Fisher Scientific, Waltham, MA, United States). The V3–V4 regions of the 16S rRNA gene were amplified and sequenced using the Illumina NovaSeq platform (Illumina, San Diego, CA, United States) by BioTree Technology Co., Ltd. (Shanghai, China). Afterward, Quantitative Insights into Microbial Ecology 2 (QIIME2, version 2019. 7) was used to process the sequencing data. Moreover, QIIME2 software annotated the species using the Silva database (Silva_123, http://www.drive5.com/usearch/manual/sintax_downloads.html). The absolute abundance of amplicon sequence variants was normalized using a standard sequence number corresponding to the sample with the least sequences. Furthermore, alpha (Shannon and Simpson indices) and beta diversities were calculated using QIIME2. Finally, LEfSe version 1.0 was used to perform the linear discriminant effect size (LEfSe) analysis with (linear discriminant analysis (LDA) score threshold four to identify differential taxa.

### 2.4 Metagenomics

Metagenomic sequencing was performed after obtaining the total DNA of the flora as described above. First, the DNA samples were fragmented to 350 bp by sonication, and then the fragments were end-repaired, A-tailed, and ligated using an Illumina adapter. After validating the library, Illumina PE150 sequencing was performed by pooling different libraries according to the requirements of effective concentration and target data volume.

Furthermore, Readfq (version 8, https://github.com/cjfields/readfq) was used to preprocess the raw data obtained from the Illumina NovaSeq 6,000 platform; the resulting clean data were used for subsequent analysis. SOAPdenova (version 2.04, http://soap.genomics.org.cn/soapdenovo.html) was used for assembly analysis of the clean data, and MetaGeneMark (version 2.10, http://topaz.gatech.edu/GeneMark/) and Bowtie2 (Bowtie2.2.4) were used for gene prediction and abundance analysis of the clean data. DIAMOND (v0.9.9.110, https://github.com/bbuchfink/diamond/) was used for species and function annotations based on the NR (version 2018-01-02, https://www.ncbi.nlm.nih.gov/) and Kyoto Encyclopedia of Genes and Genomes (KEGG) databases (version 2018-01-01, http://www.kegg.jp/kegg/), respectively. Finally, non-metric multidimensional scaling (NMDS) reduction analysis was performed using the R package (R vegan package, version 2.15.3), and LEfSe analysis (LDA score threshold four was performed using LEfSe software (version 1.0).

### 2.5 Non-targeted metabolomics

Metabolites were extracted from mouse serum samples and proportionally added to the extraction solution (methanol containing an isotopically labeled internal standard mixture), and quality control samples were prepared. The non-targeted metabolomics was then chromatographically separated by a Waters ACQUITY UPLC HSS T3 (2.1 mm × 100 mm, 1.8 μm) liquid chromatographic column and a Vanquish Ultra-high Performance Liquid Chromatograph (UPLC) (Thermo Fisher Scientific). A QE HFX mass spectrometer was used because it can acquire tandem mass spectrometry (MS/MS) spectra in an information-dependent acquisition mode controlled by acquisition software (Xcalibur, Thermo). After converting the raw data to mzXML format using ProteoWizard, the peak identification, extraction, alignment, and integration were processed using the R program package (XCMS) and matched against a self-constructed secondary mass spectrometry database in BiotreeDB (version 2.1) for material annotation. The R package was also used for non-targeted metabolomics data analysis, including principal component analyses (PCA) and orthogonal partial least squares discriminant analyses (OPLS-DA). The card value standard for metabolite screening was VIP >1, and the *p*-value of the Student’s t-test was <0.05. Lastly, the differential metabolites were annotated using the KEGG functional database, and the differential abundance score was analyzed.

### 2.6 Liver tissue RNA-seq

RNA was extracted from mouse liver tissues and analyzed for RNA integrity and total amount using an Agilent 2,100 Bioanalyzer. Then, mRNAs with poly (A) tails were enriched using oligo (dT) magnetic beads following standard procedures for transcriptome experiments. Illumina NovaSeq 6,000 was used to sequence different libraries by pooling according to effective concentration and target data volume requirements, and 150 bp paired-end readings were generated.

HISAT2 (version 2.0.5) was used to construct the reference genome index, and feature counts (1.5.0-p3) were compared using featureCounts (version 2.0.5) to measure gene expression. DESeq2 (version 1.20.0) was used to analyze the differences between the two comparison combinations. The Benjamini and Hochberg’s method was used to adjust the resulting *p*-value (Padj) to control the error discovery rate. Padj <0.05, and |log2 (fold change)| = 1 were set as the thresholds for significant differential expression. Finally, statistical enrichment of differentially expressed genes in the KEGG pathway was performed using the clusterprofiler package(version 3.8.1).

### 2.7 Real-time qPCR

Total RNAs were extracted as described previously (Sugahara and Pascual-Anaya, 2023) and qualified for subsequent qPCR experiments. Real-time qPCR was performed using the SYBR Green Premix Pro Taq HS qPCR Kit (Takara, Beijing, China) in a Real‐time PCR Instrument (Analytik Jena, Germany). The relative mRNA levels were normalized to housekeeping gene β-Actin. The 2^−ΔΔCt^ method was used to calculate the relative quantitation.

### 2.8 Liver morphological observation

Liver harvested at the eighth week in different groups were processed to observe the morphological changes. Five micron thick formalin fixed paraffin embedded sections were stained with standard Hematoxylin and Eosin stain (H&E).

### 2.9 Multi-omics association analysis

Key gut microbes, metabolites, and genes were screened, and Spearman’s correlation was used to analyze the gut microbiome-metabolite and metabolite-gene correlations, displayed using heatmaps and Sankey diagrams.

### 2.10 Pharmaceutical analysis of Chaihu Shugan

The active metabolites of Chaihu Shugan and those absorbed in the serum/liver were identified and analyzed using liquid chromatography (LC) and MS. An extraction solution (500 μL, methanol: water = 4:1; internal standard concentration = 10 μg/mL) was added to 100 mg of Chaihu Shugan to extract the active metabolites. To determine the absorbed metabolites in serum, 400 μL of serum sample was added to 40 μL hydrochloric acid (2 mol/L), centrifuged, and the supernatant was collected for testing. Hydrochloric acid (2 mol/L) was added to homogenized liver samples processed using a magnetic beads for metabolite extraction, and quality control samples were prepared for quality control.

Vanquish (Thermo Fisher Scientific) was separated by UPLC using UPLC BEH C18 column (1.7 μm × 2.1 × 100 mm) with a sample volume of 5 μL; 0.1% formic acid was added to both A and B phases. Finally, the Q Exactive Focus mass spectrometer was used to collect primary and secondary MS data; import the original mass spectrum using XCMS software; perform retention time correction; identify, extract, integrate, and align peaks; and identify the material of the peaks containing MS/MS data using a self-built secondary mass spectrum database and a corresponding fragmentation pattern matching method.

### 2.11 Network pharmacology analysis

The action targets of metabolites were identified from the ChEMBL (https://www.ebi.ac.uk/chembl/) and Traditional Chinese Medicine on Immuno-Oncology (TCMIO) databases, based on the results of active metabolites entering the blood/liver tissue identified in http://tcmio.xielab.net. The metabolites were screened and selected according to absorptivity and drug-like properties. Percent human oral absorption is ≥30%, drug-like properties are ≥0.18, and disease treatment targets can be found in the therapeutic target database (TTD) and Disease Gene Network (DisGeNET) database. A protein-protein interaction network was constructed using the database function gene ontology (http://www.geneontology.org/), KEGG (www.kegg.jp/kegg/pathway.html) enrichment of function analysis, and the STRING database (v11, stringdb.org).

## 3 Results

### 3.1 Chaihu Shugan prevents lithogenic diet-induced CGS formation

Mice in the CGS group mostly developed grade III–V stones. There was a dose-dependent gradient effect in improving stones in the treated mice, suggesting that Chaihu Shugan can prevent lithogenic diet-induced CGS formation. The stone grade information of each group is shown in Figure 1B.

### 3.2 Dynamic changes in the gut microbiota of CGS mice at different time points and restoration of homeostasis by Chaihu Shugan

**FIGURE 2:**
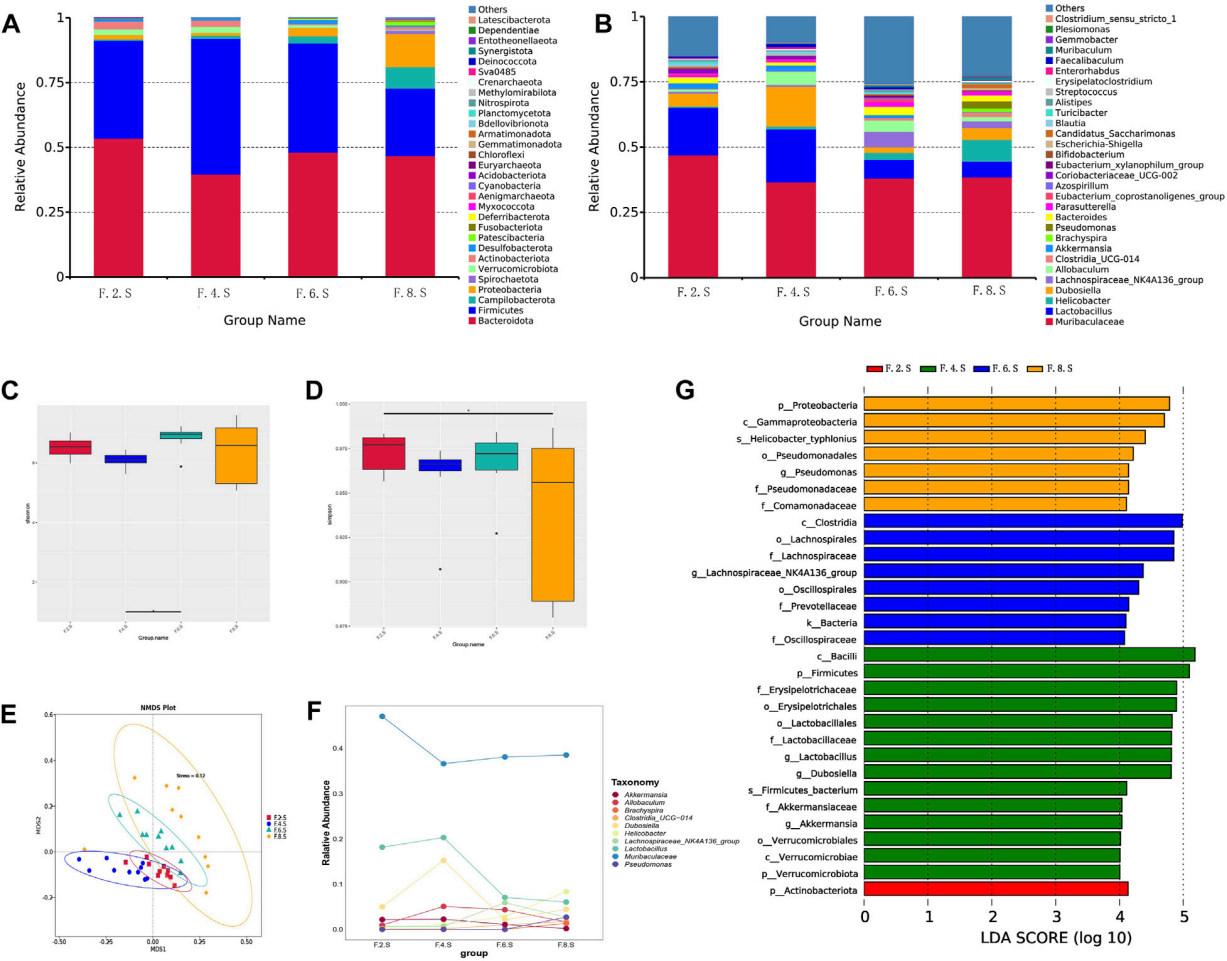
16s RNA sequencing showing dynamic imbalances in the CGS model. **(A)** Relative contributions of the top 30 phyla in each group. **(B)** Relative contributions of the top 30 genes in each group. **(C, D)** α-diversity index assessing the evenness and abundance of species (Shannon and Simpson indices). **(E)** NMDS analysis. **(F)** Line plot showing species dynamics. **(G)** LEfSe analysis was used to detect differences in the flora between the five groups (LDA >4).

Our previous unpublished studies revealed that the gut microbiota was significantly altered in patients with CGS compared with that in normal volunteers. In the present study, we observed similar but dynamic changes in the gut microbiota in the CGS mouse model. Abundance of *Bacteroidota, Firmicutes, Proteobacteria,* and *Verrucomicrobiota* changed dynamically during CGS formation at different time points (Figures 2A,B). In addition, there were no significant differences in α-diversity (Shannon and Simpson indices) except for a significant decrease in the eighth week compared to that in the second week (Figures 2C,D). Using the NMDS model based on weighted UniFrac distance and ANalysis Of SIMilarity (ANOSIM) analysis based on Bray distance, significant differences were distinguished between weeks two, four, six, and eight at different time points (Figure 2E). *Helicobacter*, *Brachyspira*, and *Pseudomonas* abundances increased in the eighth week, and *Lactobacillus* and *Akkermansia* abundances decreased (Figure 2F). LDA revealed that *Helicobacter* and Pseudomonadaceae were significantly enriched in the eighth week (Figure 2G).

**FIGURE 3:**
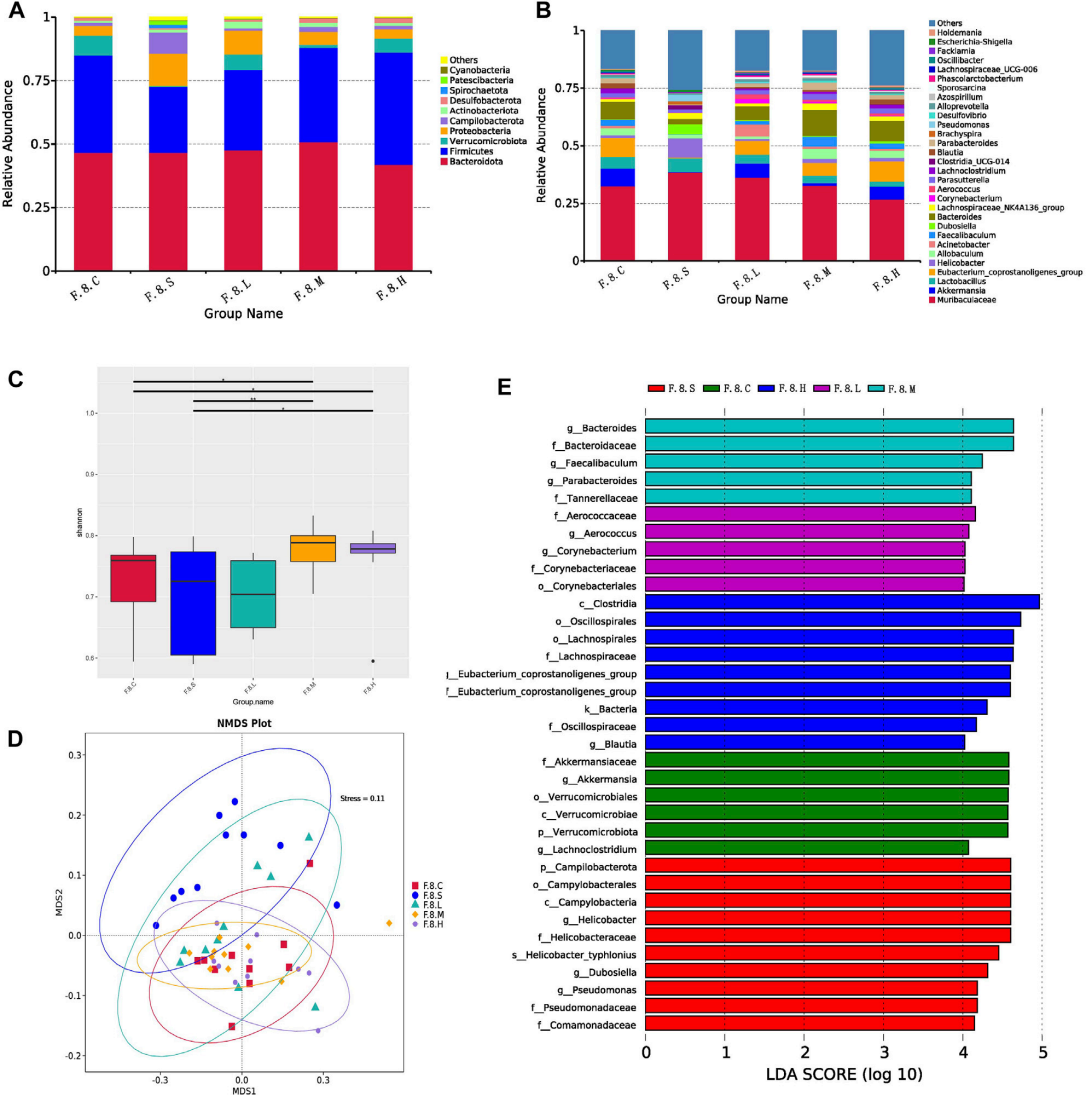
Chaihu Shugan reverses intestinal flora dysbiosis in the CGS model. **(A)** Relative contributions of the top 10 phyla in each group. **(B)** Relative contributions of the top 30 genes in each group. **(C)** α-diversity index assessing the evenness and abundance of species (Shannon indices). **(D)** NMDS analysis. **(E)** LEfSe analysis was used to detect differences in the flora between the five groups (LDA >4). Note: Group S: CGS model group. Group L: mouse with lithogenic diet and low dose of Chaihu Shugan group. Group M: mouse with lithogenic diet and medium dose of Chaihu Shugan group. Group (H) mouse with lithogenic diet and high dose of Chaihu Shugan group. Group (C) mouse with normal mouse chow.

Next, we further analyzed gut microbiota changes after the administration of Chaihu Shugan. At the genus level, there was an increase in the number of *Helicobacter*, *Brachyspira*, *Pseudomonas*, *Clostridia_UCG-014,* and *Escherichia-Shigella*. However, abundance of beneficial bacteria, such as *Akkermansia*, *Eubacterium_coprostanoligenes_group*, *Acinetobacter,* and *Faecalibaculum,* decreased (Figures 3A,B). Moreover, there was no significant difference in diversity between the experimental groups; however, there was an increase in diversity in the treatment group compared to that in the control group (Figure 3C). The NMDS model showed that the difference was clear after administration (Figure 3D). LefSe analysis (LDA >4) revealed different microbiota among various groups. Notably, most of the differential microbiotas were enriched in the CGS and treatment groups, indicating a reduction in the gut microbiota during CGS formation. Compared with that in the CGS group, the abundance of beneficial microbiota, including *Akkermansia*, *Faecalibaculum*, *Blautia*, Bacteroidaceae, and Lachnospiraceae increased in the treatment groups, while that of harmful microbiota, including *Helicobacter*, *Dubosiella*, and *Pseudomonas* decreased, suggesting that Chaihu Shugan could reconstruct the gut microbiota, especially by increasing the abundance of beneficial microbiota to prevent CGS formation (Figure 3E).

**FIGURE 4:**
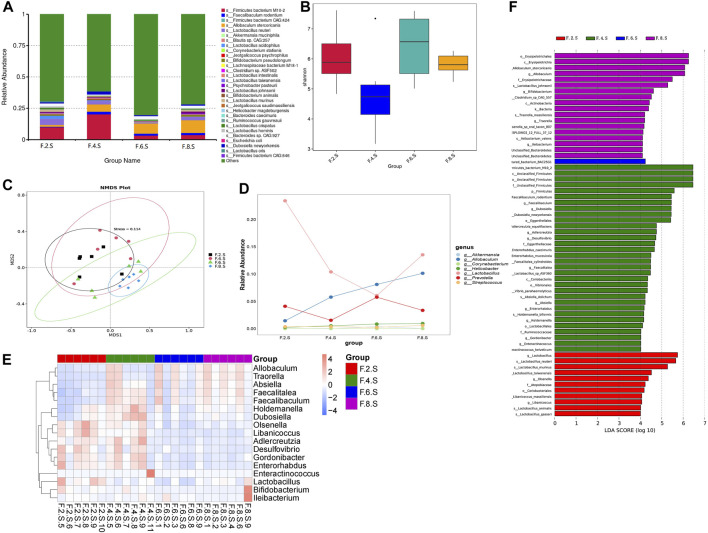
Metagenomic sequencing validates the dynamics of the gut microbiota. **(A)** Relative contributions of the top 30 species in each group. **(B)** α-diversity index assesses the evenness and abundance of species (Shannon). **(C)** NMDS analysis. **(D)** Line plots show species dynamics. **(E)** Heat maps show key flora changes. **(F)** LEfSe analysis was used to detect differences in the flora between the five groups (LDA >4).

**FIGURE 5:**
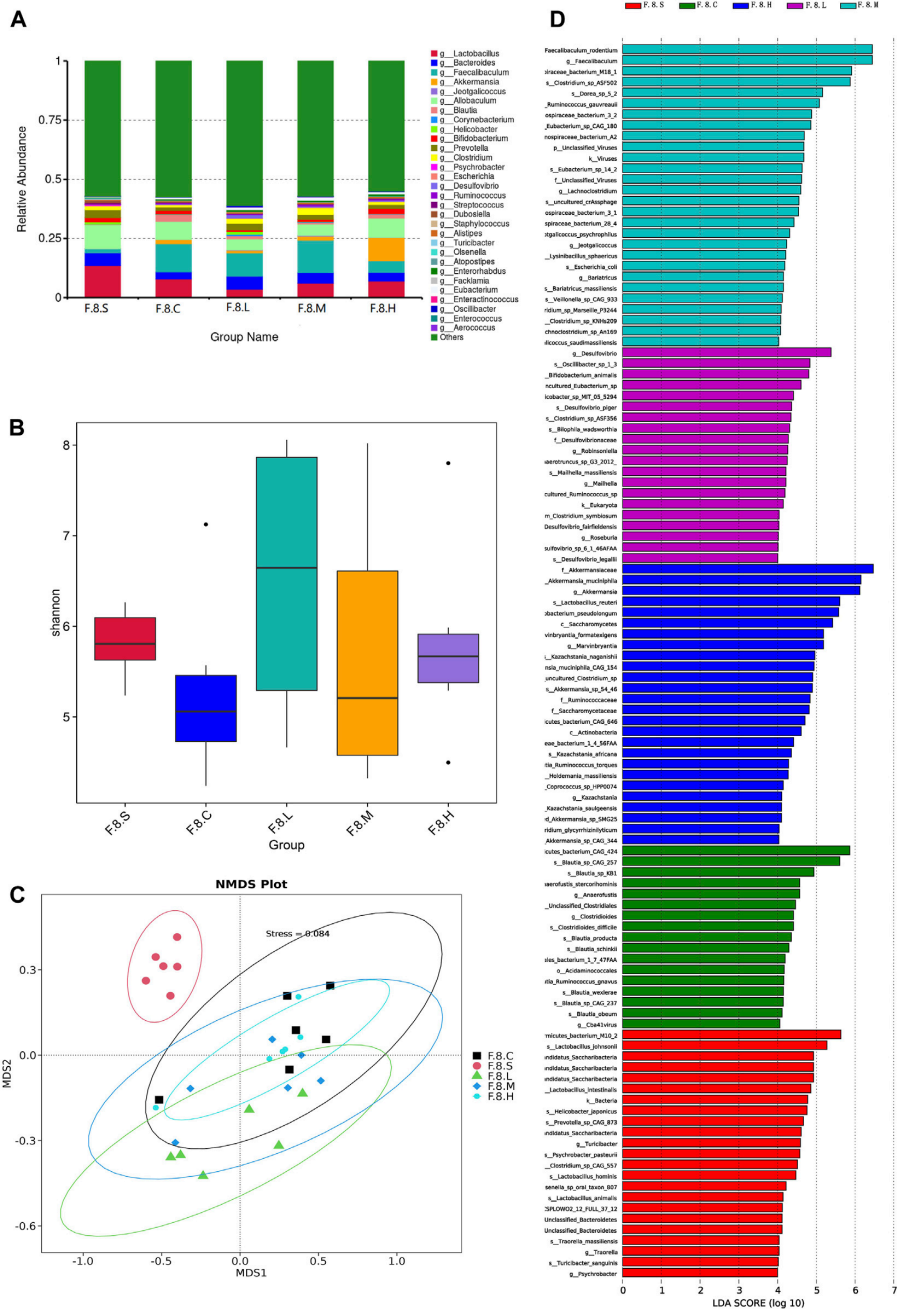
Metagenomic sequencing mining key flora of Chaihu Shugan-CGS prevention model. **(A)** Relative contributions of the top 30 species in each group. **(B)** α-diversity index assessing the evenness and abundance of species (Shannon). **(C)** NMDS analysis. **(D)** LEfSe analysis was used to detect differences in the flora between the five groups (LDA >4). Note: Group S: CGS model group. Group L: mouse with lithogenic diet and low dose of Chaihu Shugan group. Group M: mouse with lithogenic diet and medium dose of Chaihu Shugan group. Group H: mouse with lithogenic diet and high dose of Chaihu Shugan group. Group C: mouse with normal mouse chow.

Metagenomic analysis confirmed the accuracy and consistency of the 16s rRNA sequencing results (Figures 4A,B). At the genus level, the abundance of beneficial microbiota, including *g_Lactobacillus, g_Akkermansia* increased, and that of harmful microbiota, including *g_Helicobacter* and *g_Allobaculum*, decreased in the treatment groups. Dynamic NMDS clustering analysis showed significant differences among the groups and dose-dependent recovery in the treatment group (Figure 4C). The LEfSe analysis showed that *g_Allobaculum*, *s_Allobaculum_stercoricanis*, and *g_Traorella* were enriched in the CGS group, and the abundance of *g_Lactobacillus* (especially *s_Lactobacillus_reuteri* and *s_Lactobacillus_murinus*) and beneficial microbiota such as *g_Libanicoccus* was significantly decreased in the eighth week (Figures 4D–F). Treatment with Chaihu Shugan restored the abundance of beneficial bacteria, including *g_Lachnoclostridium, g_Bariatricus, s_Faecalibaculum_rodentium*, *f_*Lachnospiraceae, *g_Akkermansia, g_Roseburia,* and *s_Lactobacillus_reuteri* (Figure 5).

Based on the results of metagenomic sequencing, the changes in the gut microbiota in the CGS group were mainly characterized by a decrease in the abundance of beneficial microbiota, especially *Lactobacillus* and *Akkermansia;* Chaihu Shugan restored the gut microbiota and prevented CGS formation in a dose-dependent manner.

### 3.3 Chaihu Shugan improves lipid/amino acid metabolism and prevents CGS formation by metagenomic functional analysis

**FIGURE 6:**
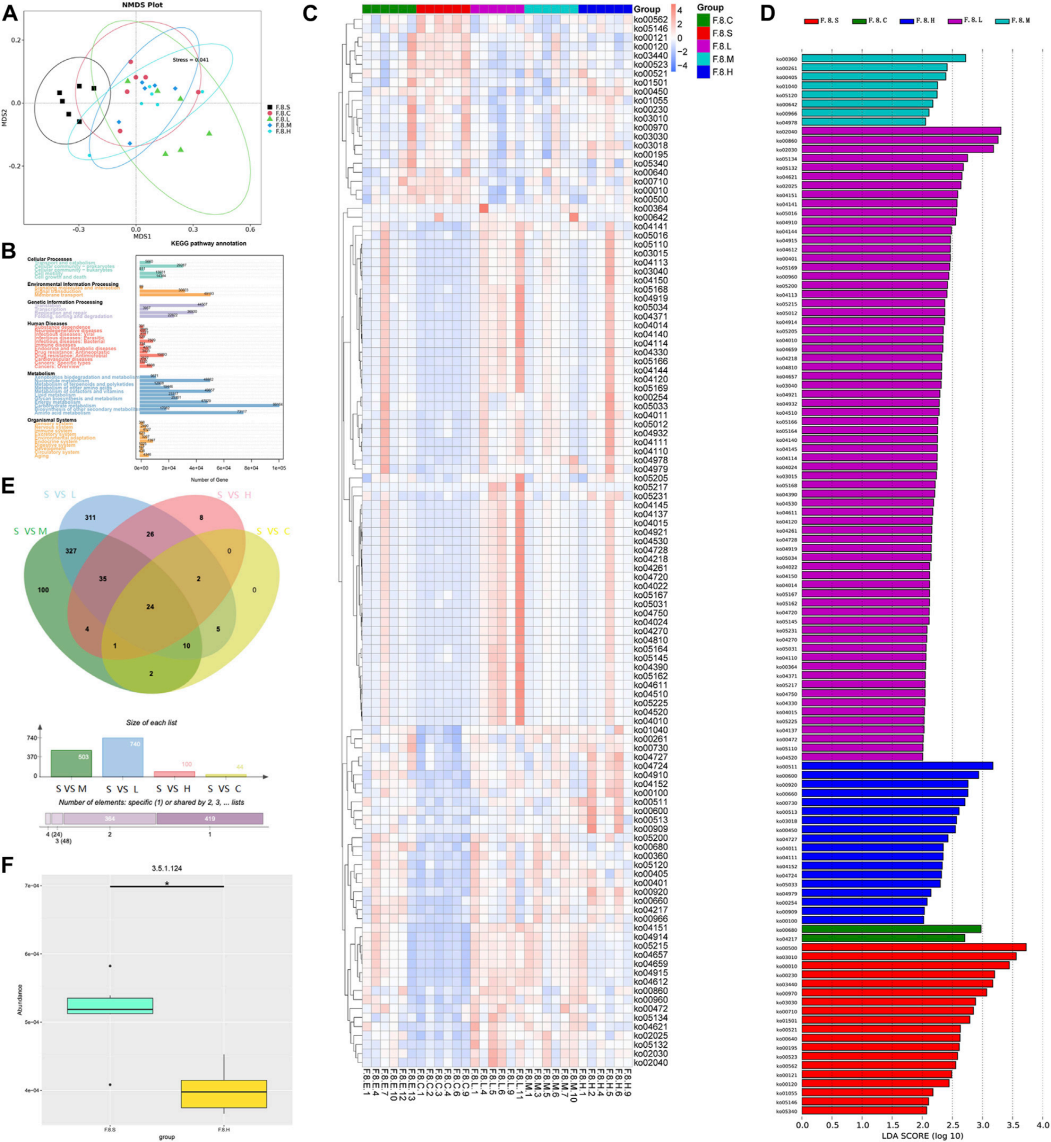
Metagenomic functional analysis mining Chaihu Shugan-CGS reversed dysfunction model. **(A)** NMDS analysis. **(B)** KEGG pathway annotation. **(C)** Heatmaps showing key functional changes. **(D)** LEfSe analysis showing key functional changes (LDA>4). **(E)** Venn diagram showing a pairwise comparison of the differences in enzyme level changes. **(F)** Histogram showing the differences in BSH between the CGS group and the high-dose Chaihu Shugan treatment group. Note: Group S: CGS model group. Group L: mouse with lithogenic diet and low dose of Chaihu Shugan group. Group M: mouse with lithogenic diet and medium dose of Chaihu Shugan group. Group H: mouse with lithogenic diet and high dose of Chaihu Shugan group. Group C: mouse with normal mouse chow.

NMDS dimension reduction analysis was conducted based on each classification level’s functional abundance using KEGG database (Figure 6A). At level 3, the functional abundance significantly differed among the five groups, especially between the CGS and control groups and between CGS and treatment groups. Based on unigene annotation results, the major genes were annotated on metabolic pathways, mainly carbohydrate, energy, and lipid metabolism (Figure 6B). Furthermore, LEfSe (LDA >2) was further used to analyze the differential functional pathways among the groups and presented with a clustering heat map. ko00121 and ko00120 (secondary and primary bile acid biosyntheses) were enriched in the CGS group. Lipid energy and amino acid metabolism (e.g., phenylalanine and sphingolipid metabolism) were enriched in the treatment group (Figures 6C,D). Moreover, we analyzed changes in levels of enzymes involved in mediating sphingolipids. Significant differences were observed in β-galactosidase metabolism, aminotransferases involved in the synthesis of amino acid metabolism, and bile salt hydrolase (BSH), which mediates the bile acid release (MetaStat analysis, *p* < 0.05). For example, BSH levels were significantly decreased after Chaihu Shugan treatment, probably caused by different microbiotas, such as *s_Faecalibaculum rodentium*, *s_Eubacterium_sp_14_*2, s*_*Lachnospiraceae*_bacterium_3_1*, and *s_*Lachnospiraceae *bacterium 28–4*. Finally, a comprehensive study revealed the dysregulation of bile acid metabolism in the CGS model. Chaihu Shugan prevented CGS formation by improving lipid, energy, and amino acid metabolism (Figures 6E,F).

### 3.4 Chaihu Shugan reversed serum dynamic metabolic changes during CGS formation by improving gut microbiota

**FIGURE 7:**
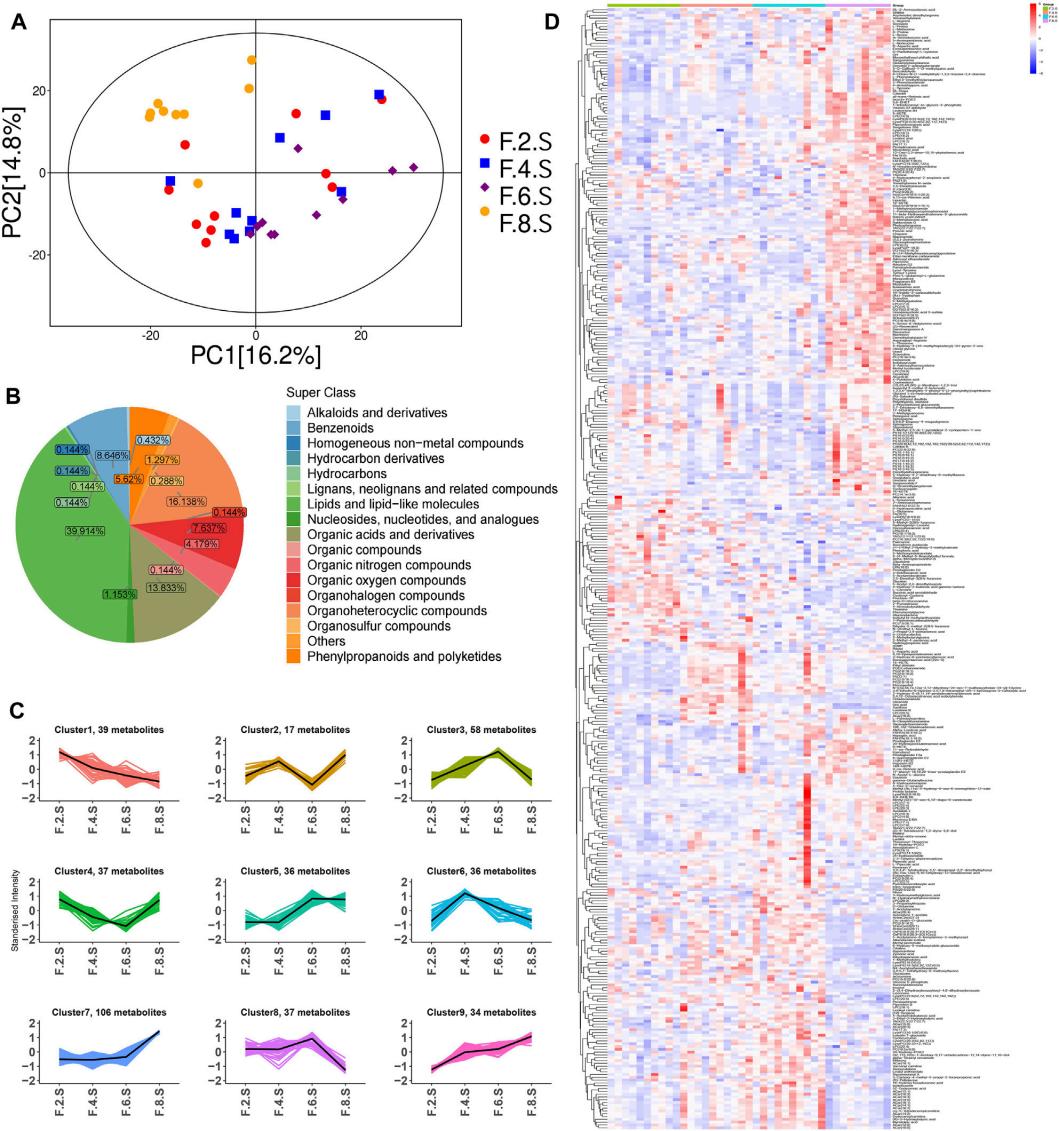
Metabolomics reveals metabolic dynamics in the CGS model. **(A)** PCA model. **(B)** Pie chart showing metabolite classification. **(C)** K-means clustering algorithm. **(D)** Heatmaps showing key differential metabolite changes.

Dynamic changes in serum metabolism were tested using LC-MS/MS. PCA showed significant variations in metabolism at different time points, especially in the eighth week (Figure 7A). During CGS formation, we identified 400 differential metabolites using analysis of variance (ANOVA) (Figures 7B,D). Furthermore, K-means clustering analysis revealed dynamic metabolic changes, including nine clusters. In cluster 1, 39 metabolites (mainly amino and fatty acids) were significantly decreased in the eighth week. In clusters 7 and 9, 109 and 34 metabolites were greatly increased in the eighth week, mainly fatty acids, glycerol phospholipids, amino acids, and fatty acyls (Figure 7C). Therefore, the formation of CGS is closely accompanied by the disturbance of lipid and amino acid metabolism.

**FIGURE 8:**
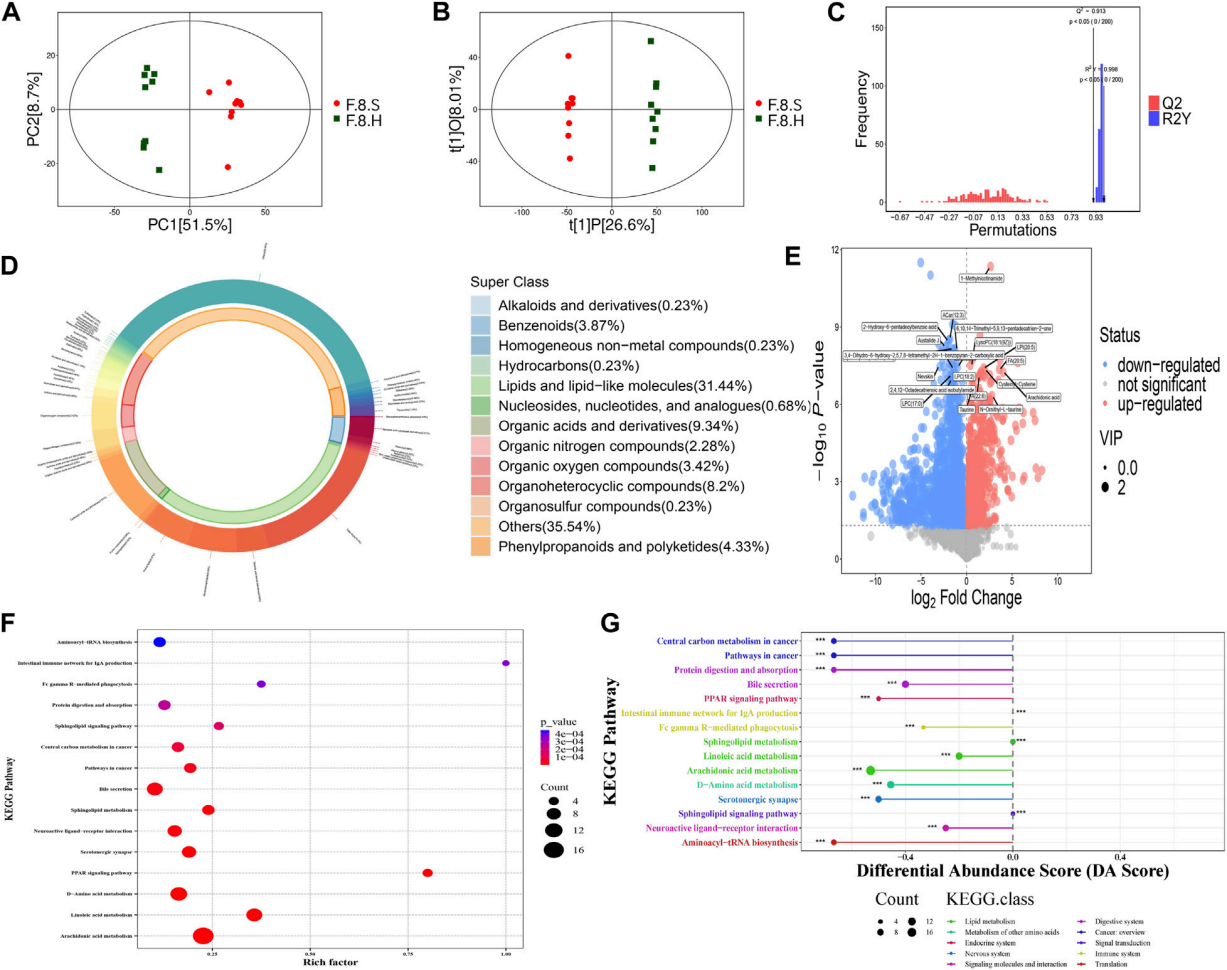
Metabolomics analysis shows that Chaihu Shugan improves metabolic dysregulation in a CGS model. **(A)** PCA model. **(B)** OPLS-DA model. **(C)** Permutation test model. **(D)** Pie chart showing metabolite classification. **(E)** Volcano plot showing key differential metabolites. **(F)** KEGG pathway analysis. **(G)** KEGG differential DA scores.

Horizontal comparisons of the metabolites in different groups were analyzed in the eighth week. Compared with the control group, the CGS group showed significant differences in terms of metabolites. Permutation replacement analysis showed that no overfitting occurred between both groups (Figures 8A–C). Furthermore, 425 differential metabolites (VIP >1, and *p* < 0.05) were identified, of which 145 decreased and 280 increased, mainly lipids and lipid-like molecules (35.06%), organic acids and derivatives (8.71%), and organoheterocyclic metabolites (8%) (Figures 8D,E). Functional pathway changes analyzed using the KEGG database showed that, compared with the control group, CGS formation was accompanied by significant metabolic disturbances, including sphingolipid, linoleic acid, d-amino acid, arachidonic acid metabolism, and bile secretion disorders (Figure 8F).

**FIGURE 9:**
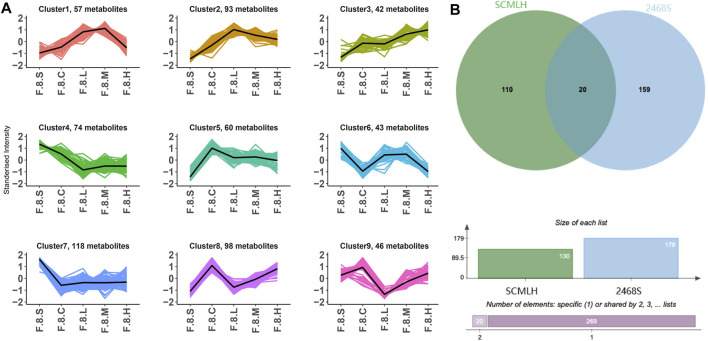
Integration of dynamic and static changes in metabolites. **(A)** K-means clustering algorithm. **(B)** Venn diagram showing the intersection of differential metabolites. Note: Group S: CGS model group. Group L: mouse with lithogenic diet and low dose of Chaihu Shugan group. Group M: mouse with lithogenic diet and medium dose of Chaihu Shugan group. Group H: mouse with lithogenic diet and high dose of Chaihu Shugan group. Group C: mouse with normal mouse chow.

Detailed comparisons of the treatment and CGS groups showed dose-dependent metabolite variations, confirming this concept. For example, 439 differential metabolites were found between the high-dose and control groups, of which 271 increased, and 168 decreased, mainly lipids and lipid-like molecules (29.01%), organic acids and derivatives (10.77%), and organoheterocyclic metabolites (10.99%). Functional pathway analysis showed that Chaihu Shugan reversed disordered metabolic pathways, including bile secretion and metabolism of linoleic acid, d-amino acid, and arachidonic acid, and restored metabolic homeostasis during CGS formation (Figure 8G). ANOVA revealed metabolite changes in different doses of Chaihu Shugan. Nine clusters were obtained using the KEGG clustering analysis. Interestingly, 43 metabolites (cluster 8) decreased, and 98 (cluster 9) increased after treatment with Chaihu Shugan in a dose-dependent manner (Figure 9A). Finally, by integrating the vertical and horizontal data, 20 differential metabolites were identified, mainly fatty and amino acids, including D-proline, stearidonic acid, 15-KETE, 11(R)-HETE, 8-HETE, lysyl-tyrosine, and tyrosyl-lysine (Figure 9B).

### 3.5 Liver transcriptome revealed changes in CGS gene function

**FIGURE 10:**
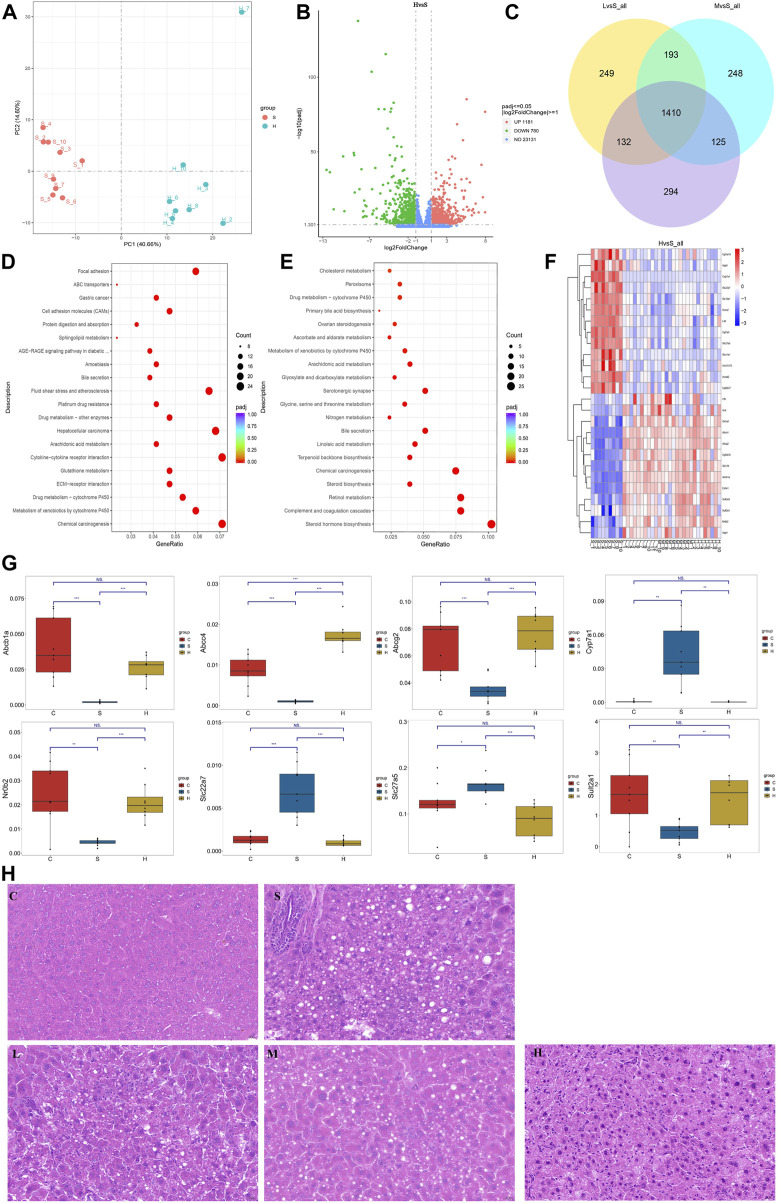
Transcriptome reveals changes in gene levels. **(A)** PCA model. **(B)** Volcano plot showing key differential genes. **(C)** Venn diagram showing the intersection of differential genes. **(D, E)** KEGG pathway analysis. **(F)** Heatmaps showing key genetic changes. **(G)** Verification of bile secretion-related genes by qPCR. **(H)** Liver steatosis in CGS model, which is ameliorated gradually after Chaihu Shugan treatments. H&E, 40x. Note: Group S: CGS model group. Group L: mouse with lithogenic diet and low dose of Chaihu Shugan group. Group M: mouse with lithogenic diet and medium dose of Chaihu Shugan group. Group H: mouse with lithogenic diet and high dose of Chaihu Shugan group. Group C: mouse with normal mouse chow.

Liver tissues were collected in the eighth week for RNA-seq to better understand how Chaihu Shugan prevents CGS formation. PCA clearly distinguished the groups, and differential gene screening was performed using DESeq2 (Love et al., 2014) (Figure 10A). The screening criteria were |log2(fold change)| = 1 and Padj (*p*-value after FDR correction) < 0.05. Compared with the control group, the CGS group had 683 upregulated and 749 downregulated differential genes. Moreover, compared with the model group, the high-dose treatment group had 1,181 upregulated and 780 downregulated differential genes (Figure 10B). The Venn diagram showed common differential genes for the three treatment groups (Figure 10C), revealing 1,410 differential genes, including 844 upregulated and 566 downregulated genes.

KEGG functional database was used to annotate functional changes in the differential genes. Compared with those in the control group, bile secretion, linoleic acid metabolism, peroxisome proliferator-activated receptor (PPAR) signaling pathway, and other secretion pathways were mainly concentrated in the CGS group (Figure 10D). Additionally, in the treatment groups, upregulated genes were mainly enriched in ABC transporters, bile secretion, arachidonic acid metabolism, and sphingolipid metabolism. Downregulated genes were mainly involved in steroid hormone biosynthesis, bile secretion, linoleic acid metabolism, arachidonic acid metabolism, primary bile acid biosynthesis, and other metabolic pathways (Figures 10E,F).

**FIGURE 11:**
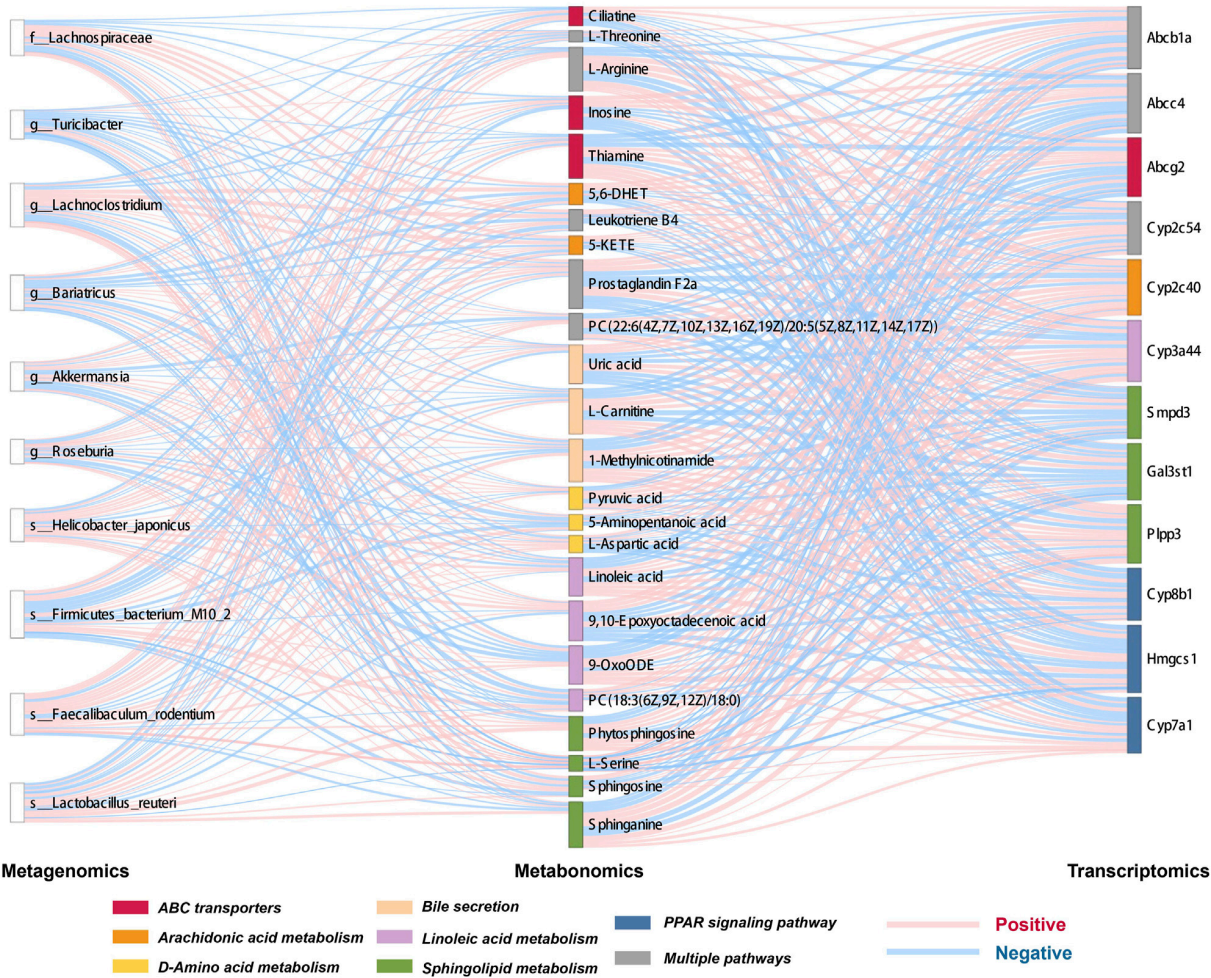
Sankey diagram showing multi-omics association analysis.

The expression levels of above differential genes were also tested by qPCR. Specifically, the levels of Abcb1a, Abcc4, Abcg2, Nr0b2 and Sult2a1, which were related to bile secretion or ABC transport, were greatly decreased when CGS performed, and recovered to different extents after high dose of Chaihu Shugan treatment. Conversely, the levels of bile acid synthesis or modification related genes, Cyp7a1, Slc22a7 and Slc27a5 were increased when CGS performed and recovered after Chaihu Shugan treatment (Figure 10G). The steatosis in the liver was obvious in the CGS model compared with control, and ameliorated gradually with the treatment of gradient doses of Chaihu Shugan (Figure 10H). Echoing this, the transcriptome results showed that Chaihu Shugan prevents CGS formation by improving the expression of genes related to lipid metabolism, bile acid metabolism, and other metabolic pathways (Figure 11).

### 3.6 Analysis of key active metabolites and network pharmacology of Chaihu Shugan

**FIGURE 12:**
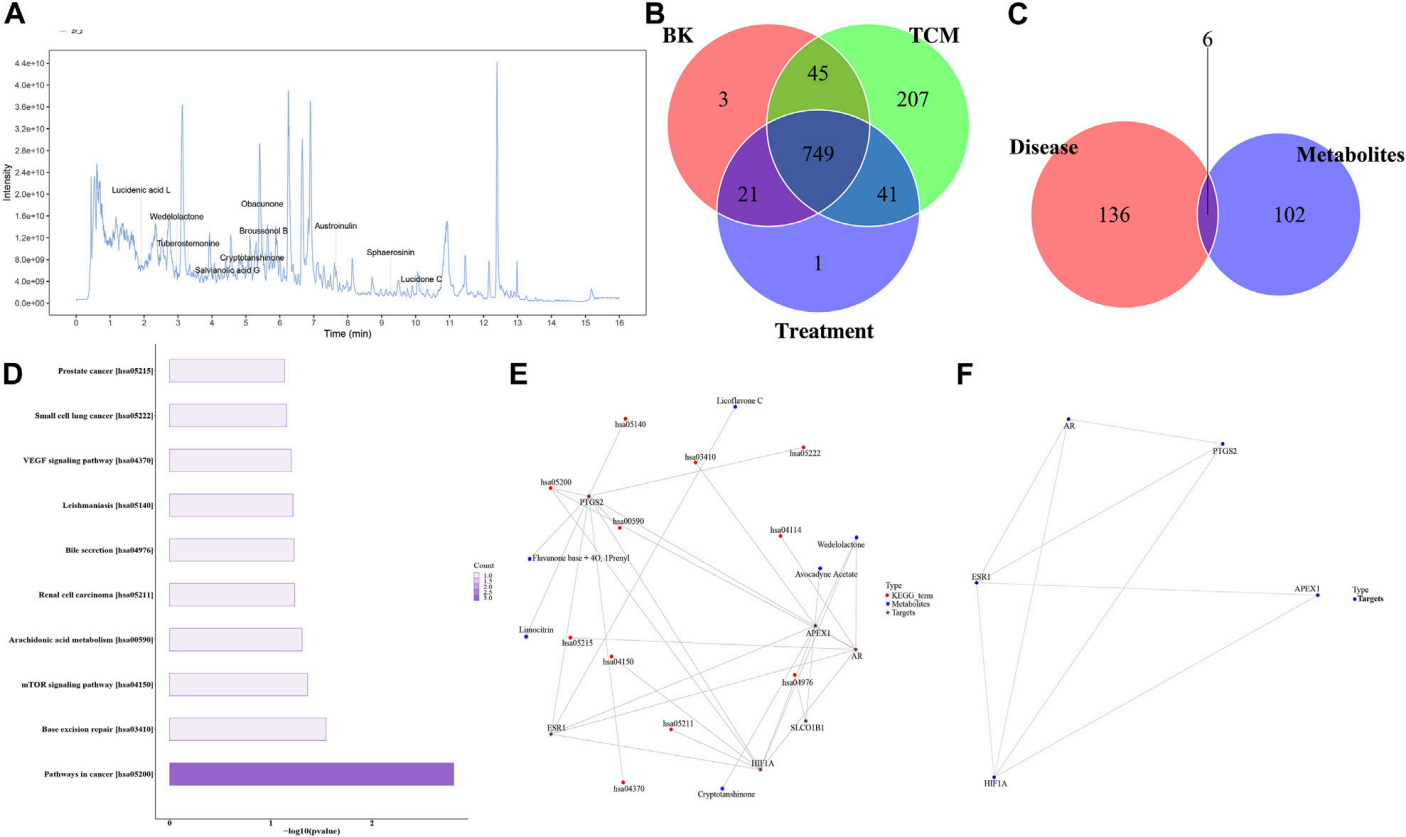
Identification of Chaihu Shugan metabolites and network pharmacological analysis. **(A)** Total ion chromatogram. **(B)** Venn diagram analysis. **(C, D)** Target protein pathway enrichment analysis histogram. **(E)** Metabolite-target-KEGG pathway interaction network diagram. **(F)** Target protein interaction network diagram.

Traditional Chinese medicine (TCM) research lacks a clear material basis. To identify the main metabolites responsible for the medicinal effect of Chaihu Shugan, LC/MS was used to determine the metabolites of the Chaihu Shugan stock solution; the mass spectrometry data obtained were matched with the TCM database (Figure 12A). We identified 1,072 secondary metabolites in Chaihu Shugan, mainly phenylpropanoids, terpenoids, terpenoids, flavonoids, and alkaloids. These active metabolites were identified in the administered serum/liver and control serum/liver, and the active metabolites were identified in combination with Chinese medicine. The prototype metabolites of Chaihu Shugan that may play an effective role in the blood/liver tissue were analyzed, of which 41 were identified (Figure 12B). Detailed prototype metabolites are listed in Supplementary Table S1. The targets of these metabolites were obtained from the ChEMBL and TCMIO databases and were screened according to their absorption rates and drug-like properties. Disease therapeutic targets were also identified from the TTD and DisGeNET database, and six potential therapeutic targets were obtained at the intersection of the two, i.e., PTGS2, ESR1, SLCO1B1, APEX1, HIF1A, and AR (Figure 12C). KEGG functional enrichment analysis showed that these targets were closely related to the mammalian target of the rapamycin signaling pathway, arachidonic acid metabolism, bile secretion, and vascular endothelial growth factor signaling pathway (Figure 12D). In addition, 34 prototype metabolites were identified in the liver tissue. Further network pharmacological analysis revealed 24 potential therapeutic targets.

Interestingly, KEGG functional enrichment analysis showed that obacunone was closely related to ABC transporters and bile secretion. We thus further analyzed the prototype metabolites of obacunone in blood and liver tissues and determined that obacunone is a common secondary active metabolite of Chaihu Shugan. Considering the similarity of its chemical structure to bile acids, obacunone might be a synthetic precursor of bile acids. Finally, Chaihu Shugan metabolite identification and network pharmacology results indicated that bile acid metabolism might be the key pathway via which Chaihu Shugan prevents CGS formation. The therapeutic effect of this single metabolite on CGS needs to be further investigated (Figures 12E,F).

### 3.7 Integration of multidimensional data to construct a ‘TCM active metabolite-gut-metabolism-gene-phenotype’ network

**FIGURE 13:**
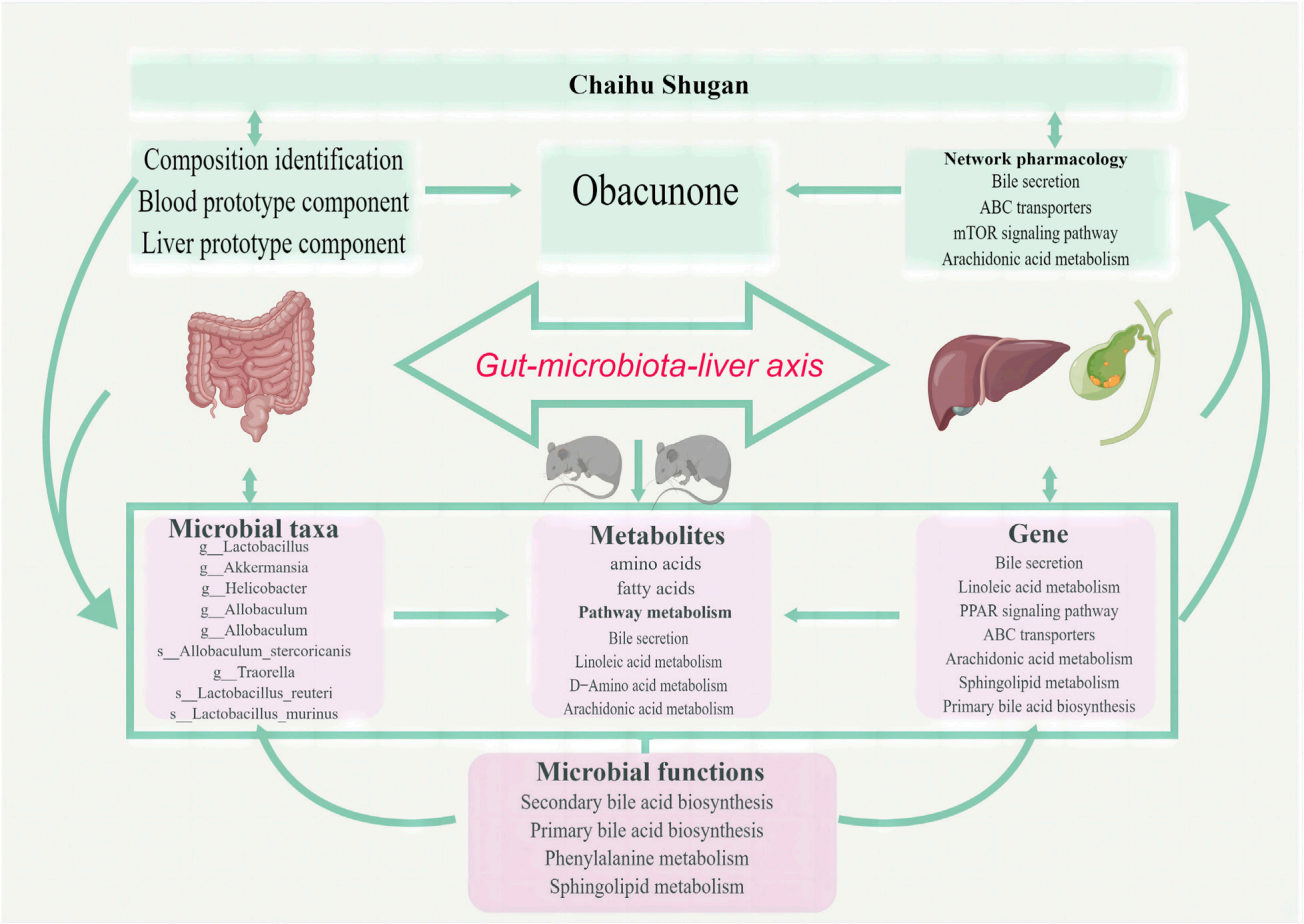
Chaihu Shugan prevents CGS formation by ameliorating the microbiota dysbiosis and metabolic disturbance.

Based on microbiome/metabolome and liver transcriptome data, obvious gut microbiota and metabolic pathway disturbances occurred during CGS formation, greatly reducing the abundance of beneficial bacteria and lipid/amino acid metabolism. Further research was conducted on Chaihu Shugan’s active metabolites. The potential key secondary metabolite, obacunone, was identified based on the analysis of the metabolites of blood/liver tissues. Based on the results of network pharmacology and multi-omics studies, we hypothesized that Chaihu Shugan restored gut microbiota homeostasis in CGS and prevented CGS formation by regulating lipid (especially bile acid) and amino acid metabolism (Figure 13).

## 4 Discussion

We investigated whether the CGS-preventive function of Chaihu Shugan by ameliorating the microbiota dysbiosis and metabolic disturbance using a multi-omics approach in a mouse model and explored potential active metabolites for drug purification and efficacy improvement.

In this study, Chaihu Shugan prevented lithogenic diet-induced CGS in a C57BL/6J mouse model dose-dependently, consistent with clinical data. In addition, several other omics assays also showed that the gut metabolism-gene axis is critical in the formation of CGS in mice, confirming our results. For example, a recent study demonstrated that gut microbiota dysbiosis can promote the CGS formation by regulating bile acid metabolism and bile secretion (Hu et al., 2022). They further showed that fecal transplantation of gut microbiota from gallstone patients to gallstone-resistant strain of mice can effectively induce gallstone formation. In another study, during CGS formation, dynamic changes in the construction and diversity of the gut microbiota occurred, characterized by an increase in the abundance of harmful microbiota (*Helicobacter, Brachyspira,* and *Pseudomonas*) and a decrease in the abundance of beneficial microbiota (*Lactobacillus* and *Akkermansia*), which is consistent with a previous study (Wu et al., 2013). Chaihu Shugan reversed this change in gut microbiota composition to that observed in the control group in a dose-dependent manner. *Lactobacillus* can prevent CGS formation by fully activating the hepatic and ileal FXR signaling pathway (Oh et al., 2021; Ye et al., 2022), and might be an active target bridge for other materials, such as *Lysimachia christinae* (Liu et al., 2021), tea (Huang et al., 2019), and coffee and caffeine (Ishizuk et al., 2003; Khochapong et al., 2021), to prevent CGS function. Moreover, *Lactobacillus* and *Akkermansia* have been reported to be associated with high daily tea consumption-induced calcium oxalate renal calculi (Chen et al., 2021). In our study, *Lactobacillus* and *Akkermansia* reduced significantly with CGS formation and recovered in the high-dose treatment group in the eighth week, indicating that *Lactobacillus* and *Akkermansia* are vital in the CGS-preventive function of Chaihu Shugan. Further functional analysis based on metagenomics showed that Chaihu Shugan improved lipid and amino acid metabolism. Several metabolism-related enzymes were altered in different groups. Levels of BSH, which regulates bile acid metabolism (Huang et al., 2019), was significantly lower in the treatment groups compared with the control group, probably induced by several differential gut microbiota, including *g_Roseburia* and *s_Lactobacillusreuteri*.

The metabolism of the CGS group underwent dynamic changes, especially in the eighth week. K-means cluster analysis showed a significant decrease in 39 metabolites, a gradual increase in 109, mainly fatty acids, glycerol phospholipids, amino acids, and fatty acyls in the eighth week. A parallel comparison of differential metabolite analysis between the CGS and control groups was performed in the eighth week, focusing on lipids and lipid-like molecules. Functional annotation analysis of both groups showed that bile secretion and sphingolipid, linoleic acid, and d-amino acid metabolism were significantly different. Interestingly, the same analysis performed on the CGS and high-dose treatment groups showed that Chaihu Shugan could reverse this alteration. As one of the possible mechanisms, the bile acid and cholesterol metabolism is influenced by hepatic FXR and CYP7A1, which are closely associated with CGS formation in the mouse model (Hu et al., 2022). Improvement in bile acid metabolism induced by sodium butyrate modulated the gut microbiota, and the FXR-FGR-15/SHP signaling pathway mitigated CGS (Ye et al., 2021). In our study, cluster analysis in different dose groups showed an obvious gradient relationship of these metabolites, confirming this basis and suggesting that Chaihu Shugan prevents CGS by restoring metabolic homeostasis.

We performed RNA-seq using liver tissue collected at the endpoint to further explore the potential mechanism of Chaihu Shugan in preventing CGS. We found that there were differences in bile secretion, linoleic acid metabolism, and the PPAR signaling pathway between the CGS and the control groups, whereas Chaihu Shugan was able to dose-dependently reverse the changes in ABC transporters, bile secretion, arachidonic acid metabolism, and sphingolipid metabolism, which is in agreement with metagenomics and metabolomics results, suggesting that Chaihu Shugan could improve lipid and bile acid metabolism and prevent CGS. It has been reported that ABC transporters are associated with multiple aspects of cholesterol metabolism, such as baseline cholesterol levels, cholesterol kinetics, hypercholesterolemia, individual responses to dietary and pharmaceutical interventions, and an increased risk of gallstones (Rudkowska and Jones, 2008; Wang et al., 2022b). Additionally, arachidonic acid (Demetz et al., 2014), linoleic acid (Azemi et al., 2022; Dumont et al., 2018; O'Reilly et al., 2020) and sphingolipid metabolism (Shin et al., 2009; Lee et al., 2010) are related to cholesterol metabolism; their pharmacological modulation could increase the hepatic expression of ABC transporters, improve bile acid/cholesterol metabolism, and reduce the risk of CGS.

Chaihu Shugan has shown remarkable efficacy in preventing CGS and restoring intestinal microbiota and metabolism, but its active metabolites are unknown. To address this issue, we performed a network pharmacology of Chaihu Shugan. LC/MS revealed that Chaihu Shugan contained 1,072 metabolites, mainly phenylpropanoids, terpenoids, flavonoids, and alkaloids. The intersection of the serum and liver metabolites revealed several potential therapeutic targets. Furthermore, KEGG functional analysis showed that ABC transporters and bile secretion were closely associated with CGS, previous microbiota and metabolism results, and other relevant studies (Figueira-Mansur et al., 2020; Ye et al., 2021; Ye et al., 2022; Zhuang et al., 2022). In our study, obacunone was the most common active metabolite in the serum, liver, and gallbladder. In a previous study, obacunone attenuated ulcerative colitis symptoms in mice by modulating the gut microbiota, disrupting the TLR4/NF-κB signaling cascades, and restoring intestinal epithelial barrier integrity (Luo et al., 2020). Ulcerative colitis increases CGS prevalence by influencing bile acids and gall bladder emptying (Mark-Christensen et al., 2017; Malik and Aurelio, 2023), where bile stasis leads to bile dehydration, increasing cholesterol concentration (Damião et al., 1997), and stone precipitation. Furthermore, obacunone prevents obesity and hyperglycemia, two independent risk factors for CGS (Lammert et al., 2016), by activating TGR5 and inhibiting PPARγ transcriptional activity (Horiba et al., 2015), which are related to bile metabolism (Chávez-Talavera et al., 2017; Jia et al., 2018; Sorrentino et al., 2020; Castellanos-Jankiewicz et al., 2021) and CGS formation (Dong et al., 2011; Wang et al., 2018; Han et al., 2019).

Overall, CGS formation was accompanied by obvious changes in lipid/amino metabolism and gut microbiota, especially a decrease in the abundance of beneficial bacteria, which was reversed by Chaihu Shugan dose-dependently. The network pharmacology analyses of Chaihu Shugan have focused on the potential target, obacunone, which is has been reported to be associated with bile acid, lipid, amino acid, and glycol metabolism, and several CGS-related metabolic diseases, including inflammatory bowel disease, obesity, and diabetes mellitus.

This study also has some limitations. Using network pharmacology analysis, we have identified several potential targets of Chaihu Shugan; however, further confirmation is needed. We have started but not yet its completed screening the identified active metabolite and their potential mechanisms, including the FXR-TGR5 signaling pathway.

## 5 Conclusion

Our analyses highlight the potential of Chaihu Shugan to prevent CGS by ameliorating the microbiota dysbiosis and metabolic disturbance. Multi-omics and correlation analyses determined the relationships between gut microbiota, metabolism, and gene alterations in the dose-dependent CGS-preventive function of Chaihu Shugan. Pharmaceutical analyses showed that obacunone might be the active metabolite responsible for this process. These findings will advance the study of Chaihu Shugan in the prevention of CGS and further provide a basis to understand the mechanism of action.

## Data Availability

The datasets presented in this study can be found in online repositories. The names of the repository/repositories and accession number(s) are as follows: https://www.ncbi.nlm.nih.gov/, PRJNA988955(16S), PRJNA989430 (Metagenomic sequencing), PRJNA991245(RNA-Seq); https://www.ebi.ac.uk/metabolights/, MTBLS7181 (Metabolomics).
